# Transcriptional and Biochemical Effects of Cadmium and Manganese on the Defense System of *Octopus vulgaris* Paralarvae

**DOI:** 10.1155/2015/437328

**Published:** 2015-01-29

**Authors:** Aldo Nicosia, Monica Salamone, Salvatore Mazzola, Angela Cuttitta

**Affiliations:** ^1^Laboratory of Molecular Ecology and Biotechnology, National Research Council, Institute for Coastal Marine Environment, UOS Capo Granitola, Torretta Granitola, 91021 Trapani, Italy; ^2^National Research Council, Institute for Coastal Marine Environment, Calata Porta di Massa, 80133 Naples, Italy

## Abstract

Due to anthropogenic activities the relative concentrations of cadmium and manganese have increased in the marine environment. Cephalopods are able to accumulate such metals and, as inhabitant of coastal waters, *Octopus vulgaris* is continuously exposed to anthropogenic activities. Since no study is available on the effects of heavy metals at molecular level in developing octopuses, herein we exposed 1-day-old paralarvae for 24 h to 10, 100, and 1000 *μ*g/L of CdCl_2_ or MnCl_2_. Cd exerted a concentration-dependent inhibition of survival and a reduction in growth rate was shown while Mn exposure did not affect the survival rate even at the highest concentrations. Gene expression profiles of *hsp70, sod, cat*, and *gst* genes were analyzed by quantitative real-time PCR and defined patterns of transcription were observed. Moreover posttranscriptional analyses were also performed suggesting the impairment of metabolic functions, under strong oxidative conditions (as occurred in paralarvae exposed to Cd) or the complete detoxification events (as occurred in paralarvae exposed to Mn).

## 1. Introduction

Cadmium (Cd) and manganese (Mn) represent, respectively, typical nonessential and essential metals for the metabolism of living organisms [1].

Cd ions which enter into cells are transported to target tissues and act by a molecular and ionic mimicry mechanism substituting the proper ions in their metabolic sites [2]. Since Cd is a permanent metal ion, it is accumulated by many organisms which determine oxidative stress, DNA damage, increase in stress proteins (hsps), and macromolecular damage [3, 4].

Cd is also a natural constituent of ocean water, with average levels between 5 and 20 ng/L in open seas [5, 6], while higher levels between 1.49 *μ*g/L in the Galician coasts [7] and 73.8 mg/L in the Dardanelles Strait [8] were reported in highly polluted coastal area.

In the last century, the massive production of Mn-containing compounds has attracted much interest [9] and now Mn is considered as an emerging contaminant [10]. Organisms need trace amounts of such metal [11]; however, exposure to high Mn levels is associated with genotoxic and cytotoxic effects affecting carcinogenesis and mutagenesis [12, 13].

Seawater typically contains approximately 2 *μ*g/L of Mn [14], depending on pH, oxygen concentration, and redox conditions and during hypoxia it reaches concentrations up to 22 mg/L [15, 16]. The toxic effects of Cd or Mn exposure have been widely described in different marine organisms; among these cephalopods are also able to accumulate essential, toxic, and nonessential elements [17, 18]. Among cephalopods,* Octopus vulgaris* is a cosmopolitan species that has been recognized as an important marine resource for the worldwide commercial fisheries [19]. The common octopus, as inhabitant of coastal waters, occupies the benthic and the pelagic zone as an adult and hatchling, respectively. Thus, it is continuously exposed to anthropogenic activities. Various studies reported data regarding the accumulation [20, 21] and the effects of metal exposure [22, 23] in tissues of adult octopuses. Although it has been shown that the developing* O. vulgaris *eggs are able to adsorb and accumulate several metals [24] and, in cephalopods, metals affect the embryogenesis, no study is available on the effects of heavy metals at molecular level in developing octopus.

In a wide variety of organisms exposure to environmental stressors such as hyperthermia, hypoxia, and heavy metals is associated with the expression of heat shock proteins (HSPs). As one of the most abundant and widely investigated families in high eukaryotes, the Hsp70 family protects the organisms from damage caused by an overload of unfolded proteins in cells [25–27]. Recently, the role of HSPs in the stresses defense system has been well documented in mollusks, such as stimulated-expression of HSP70 in* Mytilus galloprovincialis* or* Ostrea edulis* by heat shock, bacterial infection, and metal exposures [28–31]. Similarly, catalase (CAT), superoxide dismutase (SOD), and glutathione S-transferase (GST) are known to represent effective biomarkers of susceptibility to environmental contaminants. Their ability to counteract ROS increase associated with oxidative stress is essential to cellular homeostasis [32–34]. Moreover their transcriptional expression is known to be altered by metals [35–39].

To create new insights and to characterize the mechanisms involved in maintaining physiological homeostasis in response to perturbation, we have evaluated the effects of metal exposure on the octopus paralarvae. Herein we provide analysis of survival rates and morphological changes in response to metals which suggest a correlation between metal exposure and toxic effects. Additionally, a multigene/biomarker approach was used to test the effects of cadmium and manganese on the defense system of octopus paralarvae. In this scenario, the transcription of* hsp70*,* cat*,* sod,* and* gst* was profiled in response to metal exposure. Moreover, the total amount of HSP 70 protein and the activity of SOD, CAT, and GST were measured. Both transcriptional and biochemical studies suggest alterations of gene and protein expressions or enzymatic activities. Furthermore, an impairment of the metabolic capacity in ROS removal was also hypothesized after exposure to cadmium excess.

## 2. Materials and Methods 

All experiments were carried out in compliance with local laws and to date no specific permit is required for the performed experiments. Housing and husbandry of* O. vulgaris* were carried out in accordance with the best practices developed in the cephalopods community in order to optimize animal health. However, all facilities and procedures complied with the Directive 2010/63/EU.

### 2.1. Animal Sampling and General Experimental Design

*O. vulgaris* females (*n* = 4) were manually collected in the south-west coast of Sicily, over the period April-May 2012.

Octopuses were acclimated at the CNR-IAMC mesocosms (Capo Granitola, Sicily) in Millipore filtered artificial sea water (MFASW: 400 mM NaCl; 10 mM KCl; 10 mM CaCl_2_; 10 mM Hepes; pH adjusted to 8.2 with NaOH) (17 ± 1°C) and fed daily with frozen fish.

Spawning occurred 30 days after capture at a temperature of 19 ± 1°C. Egg masses were kept with the female for brooding to optimise embryo survival in MFASW (constantly aerated closed circuit, 20 ± 1°C, light/dark cycle: 12 h/12 h) until the paralarvae hatched. One-day-old paralarvae were collected, divided equally into 7 different aquaria, and exposed to MnCl_2_ and CdCl_2_ in MFASW for 24 hours, under gentle rotation at 19 ± 1°C.

Cadmium and manganese solutions were prepared using 99% of chloride salts (Sigma-Aldrich).

Treatments were performed using metal solutions at nominal concentrations corresponding to 10 *μ*g/L, 100 *μ*g/L, and 1000 *μ*g/L. Collected paralarvae were divided into 3 parts for morphological, biochemical, and gene expression analysis. Experiments were performed in triplicate.

### 2.2. Description of the Morphology

Morphological analysis was carried out using an Olympus BX50 optical microscope. Live paralarvae were sampled and anaesthetized with 0.35 mM MgCl_2_, 1 : 2 diluted with seawater. A number of 50 paralarvae per treatment were scored for mortality. Measurements of mantle (ML) and arm length (AL) were taken in both dorsal and ventral side of paralarvae. Images were analysed using NIH-Image public domain software (version 1.61 ).

### 2.3. RNA Extraction and First-Strand cDNA Synthesis

A number of 30 paralarvae per treatment were dissolved in Trizol reagent (Invitrogen Corporation, Carlsbad, USA), and further RNA purification steps were performed according to the manufacturer's instructions. RNA concentrations and quality were spectrophotometrically verified, while RNA integrity was checked using a 1.5% agarose gel. The RNA was stored at −80°C for future use. The extracted RNA (2 *μ*g) was treated with RQI RNase-Free DNase (Promega, USA) to remove any residual genomic DNA contamination, and DNase I was inactivated by adding 25 mM EDTA. First-strand cDNA was synthesised from 2 *μ*g DNase I-treated total RNA samples using oligo(dT)_18_ and Superscript III (Invitrogen Corporation, Carlsbad, USA), following the manufacturer's instructions. The cDNA mixture was stored at −20°C until needed.

### 2.4. Gene Selection and Primer Design

Genes were selected from public available database at NCBI. When not annotated, extensive BLASTP, BLASTX, and TBLASTN searches were performed to retrieve selected genes. Matching sequences were manually checked and subjected to functional ontology assignment based on Hidden Markov Models (HMMs). Selected full length sequences were reconfirmed by comparative analysis. The genes specific primer sequences, amplicon sizes, and relative GenBank Accession Number are listed in Table 1.

### 2.5. Relative Quantification Using Real-Time Quantitative Polymerase Chain Reaction (RT-qPCR)

RT-qPCR was performed using the ABIPRISM 7500 System (Applied Biosystems, Forster City, USA) with Power Sybr Green as detection chemistry (Applied Biosystems, Forster City, USA) and every reaction was repeated in triplicate. The amplification conditions were initial denaturation at 95°C for 10 min, 40 cycles of 95°C for 30 s, and 60°C for 50 s, followed by a melting curve from 60 to 95°C. Their expression stability among the different conditions was evaluated using the GeNorm software [40] and the* 18S rRNA*,* ubi,* and* tuba* were chosen as reference genes. Serial dilutions of pooled cDNAs from both control and treated samples were prepared to determine the PCR efficiency of the target and reference genes (data not shown) and amplification efficiency ranged from 1.8 to 2.1. A GeNorm normalization factor was calculated taking into account the geometric mean of the 3 selected reference genes and used to quantify the expression levels of the target genes. Relative mRNA abundances of different genes were calculated from the second derivative maximum of their respective amplification curves (Cp, calculated by triplicates). Cp values for stress-related target genes (TG) were compared to the corresponding values for a reference gene (ref.) to obtain ΔCp values (ΔCp 1/4 Cpref-CpTG).

### 2.6. Protein Extraction for HSP70 Quantification

Paralarvae (*n* = 30) homogenized in Lysis Buffer (0.5% sodium deoxycholate, 1% NP40; 0.1% SDS with PBS-T (phosphate buffered saline and 0.1% Tween-20, pH 7.5), with 2 *μ*g/*μ*L antipapain, leupeptin, and bestatin, 1 *μ*g/*μ*L aprotinin and pepstatin, 1 mM benzamidine, and 0.1 mM AEBSF). The homogenates were centrifuged to remove any insoluble debris. The supernatants were collected and dialysed against 50 mM Tris-HCl (pH 7.5). Protein concentration was determined using the Bradford assay.

### 2.7. SDS-PAGE and Western Blot

Proteins (40 *μ*g) were separated on 8% SDS-PAGE under reducing conditions. Proteins were transferred to PVDF membrane. The membrane was then blocked with 5% (w/v) nonfat milk in TBST (50 mM Tris-HCl, pH 7.5, 150 mM NaCl, and 0.1% (v/v) Tween-20 with 0.02% sodium azide) for 1 h at room temperature. Membranes were incubated over night at 4°C with the primary mouse monoclonal antibody anti-HSP70 (Sigma H5147; 1 : 500 dilution) and with the monoclonal mouse anti-actin (Sigma A2228). The membrane was washed three times with TBST and incubated with alkaline phosphatase-conjugated goat anti-mouse IgG (1 : 8000 for 1 h at r.t.). The signals from each protein band were normalized against the *β*-actin content. The Quantity One software (Bio-Rad laboratories) was used for densitometry analysis of the immunoblotted bands.

### 2.8. Protein Extraction for Oxidative Stress Evaluation

Paralarvae (*n* = 30 per treatment) were ground into fine particulate using liquid nitrogen, mortar, and pestle. The particulate from each sample was transferred to centrifuge tube filled with 5 mL of 100 mM potassium phosphate buffer pH 7.4, 150 mM KCl, and 0.1 mM PMSF and centrifuged at 10000 ×g for 10 min at 4°C. The supernatants were collected, and protein concentration was determined using the Bradford assay.

### 2.9. Determination of Antioxidant Enzyme Activities

SOD activity was measured according to McCord and Fridovich [41] and the result was expressed as units/mg protein.

CAT activity was determined according to Greenwald [42]. The results were expressed as *μ*mol min^−1 ^mg prot^−1^.

GST activity was evaluated using 1-chloro-2,4-dinitrobenzene (CDNB) as a substrate according to Habig et al. [43]. The GST activity is expressed as the *μ*mol CDNB conjugate formed mg^−1^ of total protein min^−1^.

### 2.10. Statistical Analysis

Analysis was performed in triplicate. The results were expressed as a mean value ± SD. Data were statistically analyzed by* t-*test or one-way analysis of variance (one-way ANOVA) using Statistica 6.0 (StatSoft, Tulsa, OK, USA).* P* values less than 0.05 were considered statistically significant and were indicated by ∗.

## 3. Results and Discussions

### 3.1. Survival Rate

It has been reported that in cephalopods metals are transferred from the mother to the embryos; however, cadmium and manganese seemed to be retained in the adult tissues and not transferred to the eggs [44]. Thus, to avoid any remaining contamination from collected sea water, paralarvae were exposed to metals in artificial sea water (MFSAW) for 24 h and nominal concentrations corresponding to 10 *μ*g/L, 100 *μ*g/L, and 1000 *μ*g/L of CdCl_2_ or MnCl_2_ salts were used. No lethal effect was recorded in the control group, while increased CdCl_2_ concentrations significantly affect paralarvae lethality as they exerted toxic and concentration-dependent inhibition of the survival rate (Table 2) (*P* < 0.05).

Similar results were also obtained in early life stages of different marine invertebrates including sponge, polychaete, and molluscs [45–48].

Conversely, manganese exposure did not affect the survival rate and no significant mortality was observed even at the highest concentrations (Table 2); thus, paralarvae were more sensitive to cadmium than manganese. Even if limited data on manganese cytotoxicity in marine invertebrate systems are available, our results were consistent with those in sea urchin embryos exposed to manganese [49, 50].

### 3.2. Morphological Changes

The mantle (ML) and arm length (AL) were used as indicators of growth rate (Table 2) in order to detect the presence of morphological alterations caused by metals exposure and no significant variation in AL was recorded (data not shown).

**Figure 1:**
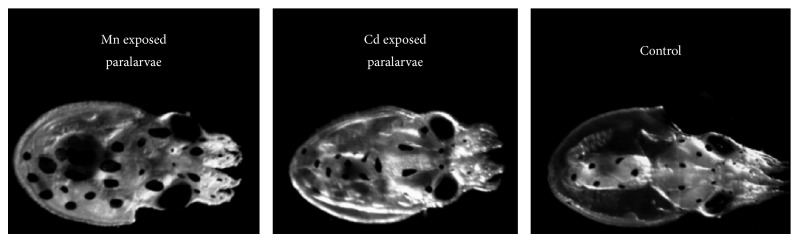
Morphological changes in response to manganese and cadmium treatments in the* O. vulgaris* paralarvae. Representative image showing morphological alterations (increase or reduction) in measurements of ML in paralarvae exposed to 1000 *μ*g/L CdCl_2_ or MnCl_2_ for 24 h.

A negative effect in growth rate was evident in paralarvae following exposure to cadmium and reduction up to 150 *μ*m in ML was found at 1000 *μ*g/L CdCl_2_ (Figure 1). These features reflect the concentration-dependent growth inhibition described previously in insect larvae of* Lymantria dispar* [51] and for some larvae of clam [48] and abalone [52]; moreover, such abnormalities became noticeable at 100 *μ*g/L CdCl_2_. Hence, this concentration would be detrimental to octopus larvae if such exposure was to occur.

Conversely, an increase in growth rate appeared following exposure to 1000 *μ*g/L MnCl_2_ and paralarvae grew about 100 *μ*m in ML. There are no studies reporting the effects on size growth in molluscs after manganese exposure; however this result is anomalous if compared with the inhibitory effects of excess manganese described in juvenile prawn and mulloway [53, 54]. It is noteworthy that such reports are confined to researches under feeding regimes. Thus, the effective growth promotion exerted by manganese in octopus paralarvae needs to be further investigated.

### 3.3. Transcriptional and Biochemical Effects of Metal Exposure on hsp70 System

The heat shock proteins have been extensively used as stress-response marker [55, 56] and their expression is known to be altered by metals [57, 58]. The high mortality rate (approximately 50%) occurred among paralarvae exposed to the highest Cd exposure (1000 *μ*g/L) and the related environmental significance prompts us to analyze transcriptional and biochemical effects exerted by Cd exclusively at 10 and 100 *μ*g/L CdCl_2_.

**Figure 2:**
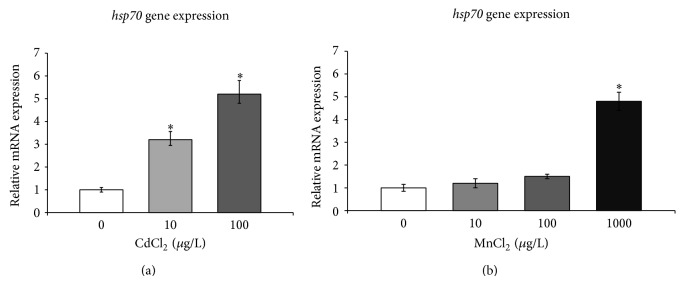
Expression of* hsp70* mRNA in response to cadmium (a) and manganese (b) treatments in the* O. vulgaris* paralarvae. The expression levels of validated internal control genes (*18S rRNA*,* ubi,* and* tuba*) were used to calculate the normalization factor in real-time PCR experiments. Values indicate the mean ± SD. Asterisks indicate statistically significant differences versus nonexposed controls at *P* < 0.05.

As hypothesised, a growth of* hsp70* mRNA expression was observed following the exposure to an increasing concentration of metals (Figure 2) and the transcript was 3.5-fold higher than that of the control group after exposure to CdCl_2_ at 10 *μ*g/L and 5.5-fold higher than that of the control group after exposure to 100 *μ*g/L. Hence, in the paralarvae of* O. vulgaris,* Cd exerted concentration-dependent effects on* hsp70 *gene expression.

Analogous results were obtained in larvae of the freshwater zebra mussel* Dreissena polymorpha* exposed to 1 *μ*g/L Cd for 24 h [59]. Furthermore,* hspA12a* a member of* Mytilus galloprovincialis* HSP70 family was upregulated after exposure to CdCl_2_ 5 and 50 *μ*g/L [28].

Few studies have reported the effects of manganese exposure on the expression of* hsp70 *inintertidal copepod* Tigriopus japonicus *[38], while its effects on* hsp70* gene expression have never been analysed in cephalopods. Herein we show that* hsp70 *overexpression (5-fold higher than that of the control group) was measured exclusively in response to massive manganese presence (Figure 2). Upregulation of* hsp*70 can be likely explained by the activation of HSF-HSP response pathway [60]. Indeed, it is well established and oxidative stress caused by pollutants can activate HSF to increase* Hsp70* gene expression [61]. Thus, in accordance with the absence of significant mortality during the treatments with Mn, it could be hypothesized that a higher threshold level of Mn is required to upregulate the* hsp70* gene expression.

Genome and transcriptome sequencing efforts revealed that most organisms including mollusks [62] possess multiple members of the Hsp70 family, some of which coexist in the same cellular compartment. Hence the expression and the overall cellular content in HSP70 are contributed by multiple isoforms codified by different genes existing in the paralarvae. Therefore to evaluate the effects of metal exposure at protein scale, we analysed the total expression of HSP70 by immunoblotting.

Densitometry analysis revealed a constitutive synthesis of the HSP70 protein in paralarvae from the control group.

Moreover, indications for a concentration-dependent synthesis of HSP70 protein was found in paralarvae exposed to Cd and Mn. Data for the cadmium-induced HSP70 response in aquatic molluscs are limited; however, in isolated gill and hepatopancreas cells of the eastern oyster,* Crassostrea virginica*, exposure to cadmium resulted in a dose-dependent increase of HSP70 proteins and in blue mussel* Mytilus edulis* HSP70 represents indicator of heavy metals accumulation [58, 63].

Similarly, herein we show that octopus paralarvae synthesize HSP70 protein, as a general protective factor, even in response to the lowest cadmium exposure.

**Figure 3:**
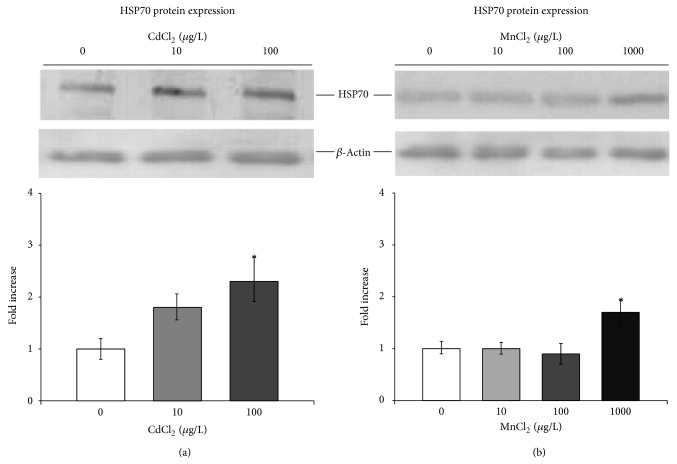
Effects of cadmium (a) and manganese (b) exposure on expression of HSP70 heat shock protein in* O. vulgaris* paralarvae. Representative immunoblot of HSP70 in paralarvae collected 24 h after exposure to CdCl_2_ and MnCl_2_ at the indicated concentrations. The histograms represent the average fold increase values ± SD of HSP70 calculated, after normalization with actin levels. Asterisks indicate statistically significant differences versus nonexposed controls at *P* < 0.05.

Analysis of HSP70 protein expression was also performed in manganese exposed paralarvae and a different profile was revealed. A mild increase in the amount of total HSP70 proteins was exclusively reported when paralarvae were subjected to 1000 *μ*g/L MnCl_2_ (Figure 3). Therefore, in octopuses, HSP70s would not respond against relative low concentrations of manganese, while it was moderately induced when the highest concentration was achieved.

In this context, the expression profile of HSP70 appears to resemble its homologue in other species including* Paracentrotus lividus* embryos [50]. Considering these results, the ubiquitous nature of HSP70 family, and activities it appears that expression of HSP70s represents a general feature of the cellular response to metals across the different taxa, including early life stages.

### 3.4. Transcriptional and Biochemical Effects of Metal Exposure on Antioxidant System

**Figure 4:**
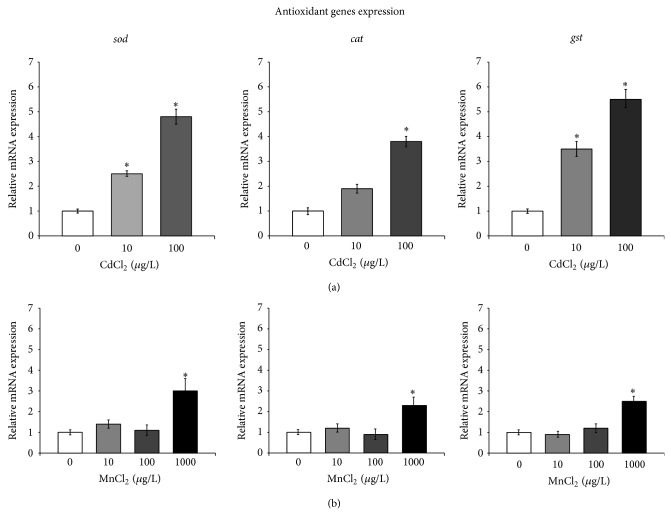
Expression of antioxidant enzymes mRNA in response to cadmium (a) and manganese (b) treatment in the* O. vulgaris* paralarvae. The expression levels of 3 validated internal control genes (*18S rRNA*,* ubi,* and* tuba*) were used to calculate the normalization factor in real-time PCR experiments. Values indicate the mean ± SD. Asterisks indicate statistically significant differences versus nonexposed controls at *P* < 0.05.

The mRNA levels of the three selected genes (*sod*,* cat, *and* gst*) potentially involved in cell stress defense and antioxidant mechanisms are shown in Figure 4.

The relative expression of* sod*,* cat, *and* gst* increased in paralarvae exposed to Cd, reaching its peak at 100 *μ*g/L CdCl_2_. In particular,* sod *expression was elevated to approximately 5-fold greater than the control,* cat* mRNA accumulated to 3.5-fold greater than the control, and* gst *transcript was overexpressed approximately 6-fold greater than the control group.

Our data correspond to other works showing the induction of antioxidant genes in fish [64, 65] exposed to Cd.

Additionally, similar results were obtained in* Crassostrea gigas* [36]. Therefore, the expression of the mRNA for antioxidant genes suggests that ROS were induced by Cd in paralarvae and that the antioxidant system was transcriptionally enhanced to remove the ROS.

The transcriptional activity of these genes, in response to Mn, did not show such a huge variation. Indeed, the relative expression of* sod*,* cat, *and* gst* peaked exclusively in paralarvae exposed to 1000 *μ*g/L MnCl_2_.

Even though there are limited data available on transcriptional effects exerted by Mn exposure, our findings are in accordance with the results from the copepods* Tigriopus japonicus* exposed to Mn [38, 66].

The analysis of mRNA levels allowed us to hypothesize also a coordinate expression of stress/oxidative metabolism-related genes. Indeed, the* sod, cat,* and* gst* mRNA levels appeared mutually correlated in paralarvae exposed to both metals, a finding which is consistent with their relatively similar response to metal treatments.

These genes are under the control of antioxidant response elements (AREs) in many organisms [67], and their coordinate expression may be associated with detoxification events in response to variations of the redox status. Thus, it could be hypothesized that putative transcription factors coregulating these genes (likely through ARE or similar controlling elements in their promoter sequences) are already present at the larval stage.

**Figure 5:**
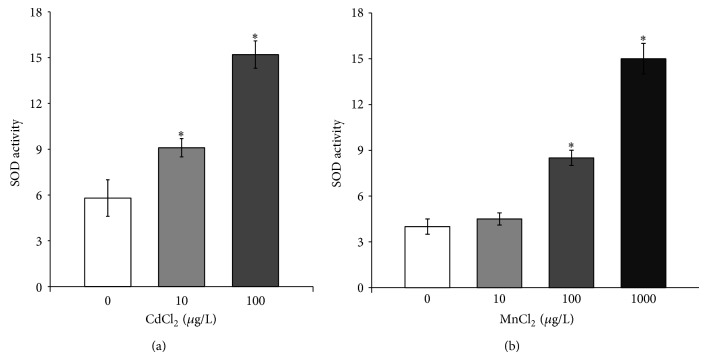
Superoxide dismutase activity in* O. vulgaris* paralarvae exposed to cadmium (a) and manganese (b). Paralarvae were treated with 0 (control), 10, and 100 *μ*g/L CdCl_2_ or 10, 100, and 1000 *μ*g/L MnCl_2_ for 24 hours. All data are represented as mean ± SD. Asterisks indicate statistically significant differences versus nonexposed controls at *P* < 0.05.

The activity of SOD, CAT, and GST enzymes was also profiled in order to evaluate the general status of antioxidant system and to test the presumptive impairment of the metabolic capacity. The SOD activity increased in paralarvae collected after 10 *μ*g/L and 100 *μ*g/L CdCl_2_ exposure (Figure 5(a)). Conversely, increasing in SOD activity was found in paralarvae after exposure to 100 and 1000 *μ*g/L MnCl_2_ (Figure 5(b)).

**Figure 6:**
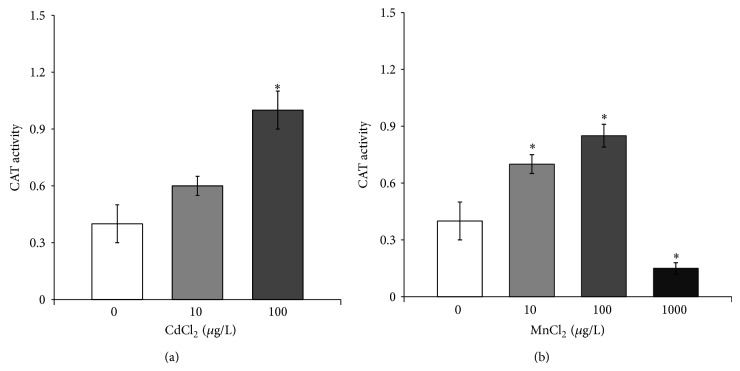
Catalase activity in* O. vulgaris* paralarvae exposed to cadmium (a) and manganese (b). Paralarvae were treated with 0 (control), 10, and 100 *μ*g/L CdCl_2_ or 10, 100, and 1000 *μ*g/L MnCl_2_ for 24 hours. All data are represented as mean ± SD (30 paralarvae per treatment). Asterisks indicate statistically significant differences versus nonexposed controls at *P* < 0.05.

Concerning the CAT activity, a dose-dependent increase was found in paralarvae, collected after 10 and 100 *μ*g/L CdCl_2_ treatments (Figure 6(a)). MnCl_2_ exposure differently affected the CAT activity; indeed, it was considerably higher compared to controlsin response to 10 *μ*g/L and 100 *μ*g/L MnCl_2_ exposure, while it dropped markedly in paralarvae exposed to 1000 *μ*g/L MnCl_2_ (Figure 6(b)).

**Figure 7:**
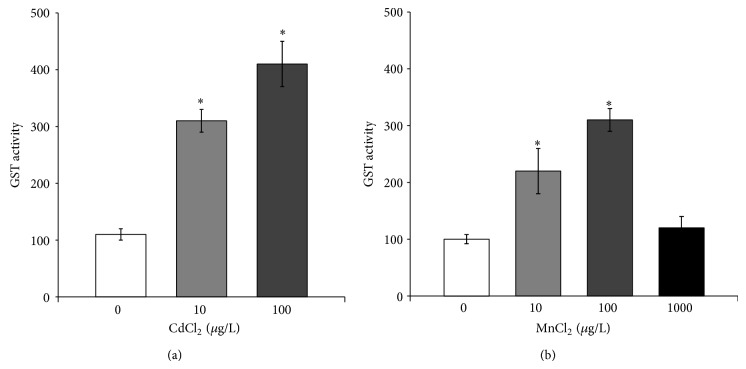
Glutathione S-transferases activity in* O. vulgaris* paralarvae exposed to cadmium (a) and manganese (b). Paralarvae were treated with 0 (control), 10, and 100 *μ*g/L CdCl_2_ or 10, 100, and 1000 *μ*g/L MnCl_2_ for 24 hours. All data are represented as mean ± SD. Asterisks indicate statistically significant differences versus nonexposed controls at *P* < 0.05.

Lastly, GST activity was similarly affected by CdCl_2_ and MnCl_2_ since it increased in response to 10 *μ*g/L and 100 *μ*g/L, while a decrease was measured in paralarvae exposed to 1000 *μ*g/L MnCl_2_ (Figure 7).

Considering the production of ROS caused by metal exposure, the activity of antioxidant enzymes is usually enhanced [32, 68, 69]. Thus it is reasonable to hypothesize that in octopus paralarvae the activity of antioxidant enzymes was enhanced to restore physiological homeostasis. At relatively low or medium metal concentrations, the ROS production in paralarvae may be nullified by the increased SOD, CAT, and GST activities and it may be considered as a general protective strategy to avoid toxicity.

Conversely, metal excess resulted in an antioxidant activities reduction or inhibition. A reduction in antioxidant activities was also reported in early life stages of several organisms [70, 71] after exposure to metal stress.

Thus, even if the defense mechanisms are activated, the huge toxicity exerted by cadmium beyond a certain level (1000 *μ*g/L) results in a decrease of antioxidant function (data not shown) that is harmful to the survival of paralarvae and may be associated with an impairment of metabolic capacity.

Additionally low levels of antioxidant enzymes in mussels have been interpreted as a sign of susceptibility to oxidative stress [72, 73].

Therefore, we deduced that the antioxidant dropping off may be associated with complete detoxification events (as likely occurred in paralarvae exposed to Mn) or with the impairment of metabolic functions under strong oxidative stress which in turn resulted in the inability to restore the physiological homeostasis (as occurred in paralarvae exposed to Cd).

## 4. Conclusions

*O. vulgaris* is one of the most economically valuable species among cephalopods and the intake of essential and nonessential elements from the increasing consumption of octopus flesh may represent a potential risk of human exposure.

In the present study, concentrations from 10 to 1000 *μ*g/L of both metals were used; they were selected and used as they likely represent environmentally relevant, albeit elevated, exposure. Moreover the concentrations herein used are similar to those tested in other studies [37, 38, 74, 75].

Data herein presented strongly suggest a different tolerance of the paralarvae towards cadmium and manganese. Cadmium has an oxidative stress potential greater than manganese which resulted in hazardous effect and mortality also at relatively low concentrations. Additionally, to the best of our knowledge herein we provide the first effort towards the depicting and understanding of the transcriptional and biochemical mechanisms of the response against metal exposures in the juvenile octopuses. Moreover, this work represents the first report on the effects of Mn in molluscs. Our results indicated that Cd and Mn exposure could affect stress and detoxification related pathways at gene expression, protein, and enzymatic level in* O. vulgaris*.

However the expression profile analysis of antioxidative stress genes in paralarvae needs to be further investigated. Thus a comprehensive mining and profiling of the* hsp70*,* sod, cat, *and* gst* gene families could provide a better understanding of the molecular mechanisms of metal-induced cellular damage in juvenile octopus.

## Figures and Tables

**Table 1:** Oligonucleotide primers used in this study.

Gene	Primers	Sequences (5′-3′)	Size (bp)	GenBank
*hsp70 *	hsp70-F	CCCATAGTTAAGGGGTTGACATC	197	JR438844.1
	hsp70-R	GTGCTTGTTGGTGGCTCTACTAG		

*sod *	sod-F	AGCACTCACGCATCCATTAG	156	JR437853.1
	sod-R	GGGTCTTCCGAATCTGTTTC		

*cat *	cat-F	CCGTCCCTTTGATAGTTGG	114	JR448615.1
	cat-R	GGGTCGCCTGTATTCCTAC		

*gst *	gst-F	AACCCAAATTCCCCAGAGTAT	129	X65543.1
	gst-R	GTCGTCCAAGATGTCATAGAAGC		

*tubA *	tuba-F	ACTGGTGTCCAACTGGCTTC	105	X15845
	tuba-R	TGCTTAACATGCACACAGCA		

*actB *	act-F	TGATGGCCAAGTTATCACCA	103	AB053937
	act-R	TGGTCTCATGGATACCAGCA		

*18S *	18S-F	AGTTCCGACCGTAAACGATG	142	FJ617439
	18S-R	CCCTTCCGTCAATTCCTTTA		

*ubi *	ubi-F	TCAAAACCGCCAACTTAACC	113	FJ617440
	ubi-R	CCTTCATTTGGTCCTTCGTC		

**Table 2:** Survival (S) expressed as percentage of alive paralarvae and morphological alteration (ML), expressed as mantle length, of exposed paralarvae.

Level (*μ*g/L)	CdCl_2_		MnCl_2_	
	S (%)	ML (mm)	S (%)	ML (mm)
0	99 ± 2	1.55 ± 0.03	98 ± 1	1.55 ± 0.03
10	84 ± 7^*^	1.57 ± 0.02	99 ± 1	1.55 ± 0.05
100	75 ± 11^*^	1.51 ± 0.02	94 ± 5	1.57 ± 0.02
1000	53 ± 8^*^	1.40 ± 0.03^*^	97 ± 2	1.65 ± 0.03^*^

Data are expressed as mean values ± standard error (*n* = 3). Asterisks indicate significant differences with respect to control values at *P* < 0.05.
